# Goat uromodulin promoter directs kidney-specific expression of GFP gene in transgenic mice

**DOI:** 10.1186/1472-6750-5-9

**Published:** 2005-04-11

**Authors:** Yue-Jin Huang, Nathalie Chretien, Annie S Bilodeau, Jiang Feng Zhou, Anthoula Lazaris, Costas N Karatzas

**Affiliations:** 1 PharmAthene Canada Inc. (formerly Nexia Biotechnologies Inc.), 1000 St-Charles Avenue Block B, Vaudreuil-Dorion, QC J7V 8P5, Canada; 2 Current address: Genomatix Corporation, 119 Norfolk Ave SW, Roanoke, VA 24011, USA; 3 Current address: Quebec Transgenic Research Network, McGill University, 1110 Ave Pine West, Montreal, QC H3A 1A3, Canada

## Abstract

**Background:**

Uromodulin is the most abundant protein found in the urine of mammals. In an effort to utilize the uromodulin promoter in order to target recombinant proteins in the urine of transgenic animals we have cloned a goat uromodulin gene promoter fragment (GUM promoter) and used it to drive expression of GFP in the kidney of transgenic mice.

**Results:**

The GUM-GFP cassette was constructed and transgenic mice were generated in order to study the promoter's tissue specificity, the GFP kidney specific expression and its subcellular distribution. Tissues collected from three GUM-GFP transgenic mouse lines, and analyzed for the presence of GFP by Western blotting and fluorescence confirmed that the GUM promoter drove expression of GFP specifically in the kidney. More specifically, by using immuno-histochemistry analysis of kidney sections, we demonstrated that GFP expression was co-localized, with endogenous uromodulin protein, in the epithelial cells of the thick ascending limbs (TAL) of Henle's loop and the early distal convoluted tubule in the kidney.

**Conclusion:**

The goat uromodulin promoter is capable of driving recombinant protein expression in the kidney of transgenic mice. The goat promoter fragment cloned may be a useful tool in targeting proteins or oncogenes in the kidney of mammals.

## Background

Uromodulin is the most abundant protein in the urine of all placental mammals, with approximately 50–200 mg released per day. It is an 85-kD glycosylphosphati-dylinositol (GPI)-anchored glycoprotein secreted from the epithelial cells of the thick ascending limbs (TAL) of Henle's loop and the early distal convoluted tubule in kidney [1]. Uromodulin has an identical amino acid sequence, immunologic cross-reactivity and tissue localization as Tamm-Horsfall protein (THP) [2]. Physiological functions of uromodulin have remained elusive, but recent knock-out studies have suggested that it plays a role in defense against urinary tract infection [3,4]. It may also have an immuno-suppressive role [5]. The uromodulin gene promoter has been cloned from human, bovine, rat and mice species [6-8]. The abundance of the uromodulin protein in urine makes the uromodulin promoter a good candidate for driving the production of recombinant (rc)-proteins in the kidney and eventual excretion into the urine of transgenic animals (mice, goats, etc.). The potential of targeting rc-proteins in the urine may have advantages over the more widely used mammary system. Rc-protein may be harvested right after birth for both sexes from a rather simple medium. However, for such a system to be effective, expression levels should be in the range of 0.5–1 g/L to satisfy commercial applications. To date hGH is targeted into mouse urine using mouse uroplakin II at ~0.5 μg/mL [9]. The same promoter has been used to target the human granulocyte macrophage-colony stimulating factor (hGM-CSF) to the kidney of transgenic mice with the urine secretion level up to 180 ng/mL [10]. Secretion of rc-alpha1-antitrypsin in mouse urine at levels as high as 65 μg/mL has been achieved using the human uromodulin promoter [11].

As a first step in further exploring the production of rc-proteins in the urine of transgenic animals, we cloned a goat uromodulin gene promoter fragment, fused it to a GFP reporter gene and studied GFP targeting and distribution in the kidney and more specifically in the epithelial cells of the TAL of Henle's loop and the early distal convoluted tubule.

GFP is an important tool in molecular and cellular biology as a transcriptional reporter, fusion tag, or biosensor. It is considered as an almost ideal *in vivo *reporter gene, because it does not interfere with cell vitality. It is highly sensitive and it can be easily detected using fluorescence microscopy.

## Results

### Cloning and characterization of the goat uromodulin gene promoter and its partial 3' end

**Figure 1:**
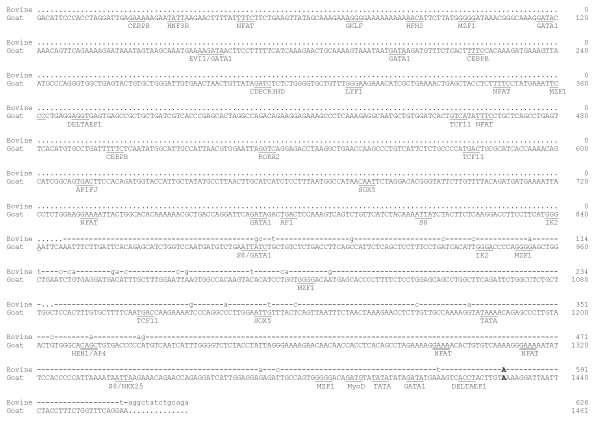
**Sequence comparison of the proximal 5' -flanking regions of goat and bovine uromodulin genes. **Identical nucleotides are indicated dashed. The putative transcriptional start site of the goat promoter (nt 1428) is in bold, deducted from the known bovine start site (nt 591, bold). TATA, CCAAT boxes and other potential binding sites of transcription factors are underlined. The nucleotide sequences of the goat uromodulin gene promoter and the goat uromodulin gene partial 3' end are available in the GenBank Database under the accession numbers AY702660 and AY702661, respectively.

A 3.7 kb fragment of the goat uromodulin gene fragment containing a 1.5 kb 5' flanking region, exon 1, intron 1, exon 2 and part of intron 2 was cloned by PCR genomic walking based on the bovine sequence [7]. The goat uromodulin promoter proximal end shares 95 % identity with bovine uromodulin gene promoter sequence [7]. The potential transcription initiation site as well as DNA transcription factor binding sites of the goat uromodulin promoter fragment was deduced by comparison with the bovine promoter sequence (Figure 1). The proximal 1.5 kb 5'-flanking region contains typical eukaryotic promoter elements including two CCAAT boxes at position -65 and position -558, a TATA box at position -30, and the conserved sequence TGTAAAAGG (nucleotides -3 to +6). The proximal 5'-flanking region also contains several putative binding sites for known transcription factors such as CEBPB, NFAT, MZF1, SOX5, TCF11, GATA1, IK2 and DELTAEF1, etc., as analyzed with MatInspector V2.2 [12]. In addition, several consensus binding sites for activator protein-1 (AP-1) are present.

A 2.7 kb fragment of the goat uromodulin gene 3' end was also cloned using PCR genomic walking and degenerate primers designed based on conserved nucleotides between bovine and human [2] uromodulin sequences. The cloned 3' end fragment contains part of exon 10, intron 10 and exon 11 including the 3' untranslated region and a potential polyadenylation site (AATAAT). Assignment of exon/intron boundaries was achieved by comparison to the bovine sequence [7].

**Figure 2:**
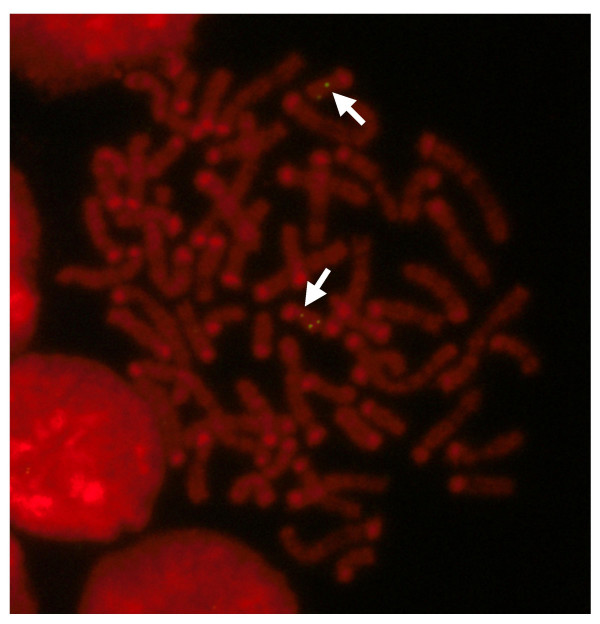
**FISH analysis of R-banded metaphase spreads from peripheral blood with a goat uromodulin promoter probe. **The arrows indicate the localization of the uromodulin gene on goat chromosome #25, whereas the signal of the BAC clone (EPO) was used for chromosome assignment.

FISH analysis, performed using the 1.5 kb fragment of the goat uromodulin promoter as a probe, revealed that the gene was localized on goat chromosome No. 25 (25q14-17). Chromosome assignment was performed using a known BAC goat genomic clone as a marker [13] (Figure 2).

### Characterization of mice transgenic for goat uromodulin-GFP construct

**Figure 3:**
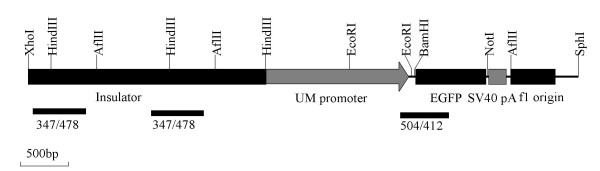
**Schematic drawing of the pING32 transgene, excised from pING32 by digestion with XhoI-SphI. **The lower bars indicate the lengths of the PCR products and the primer pairs used for the screening of the transgenic mice.

**Figure 4:**
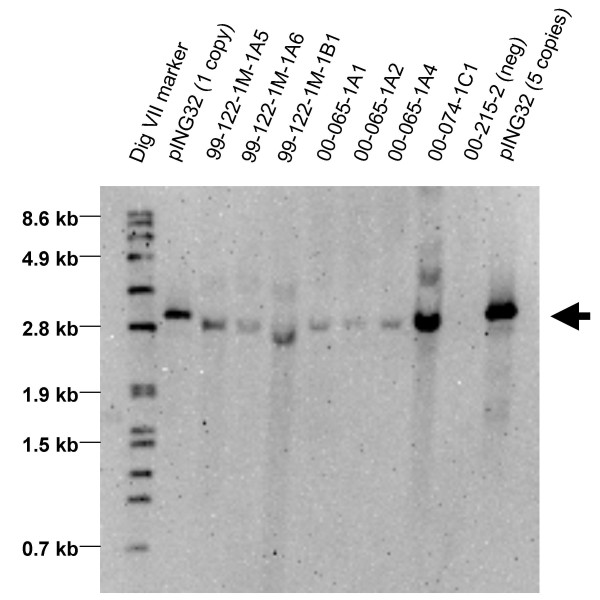
**Southern blot analysis of AflII digested mouse genomic DNA. **Lane 1: Dig-labeled Marker VII (Roche). Lane 2 & 11: GFP transgene plasmid DNA. Lane 3–9: tail DNA derived from GFP-positive mice. Lane 10: tail DNA from a non-transgenic mouse. 7 μg of DNA was loaded in each lane (lanes 3–10). The membrane was hybridized with a Dig-labeled PCR probe (Acb504-412, see Figure 3). The arrow indicates the GFP transgene.

In order to test the functionality of the cloned goat uromodulin promoter we generated transgenic mice using expression DNA cassette GUM-GFP (Figure 3) in which 2 copies of the chicken β-globin insulator sequences were fused 5' upstream to the 1.5 kb fragment of the GUM promoter. Three transgenic founder mice were mated with wild type mice and tail DNA samples from the F1 and F2 offspring were analyzed by PCR and Southern blot (Figure 4). Digestion with AflII resulted in a 2.8 kb fragment which includes 500 bp of the insulator sequence, the promoter region and the full length of the GFP cDNA. The transgene may be truncated in 99-122-1M-1B1 mouse as the major hybridizing band appeared smaller in size (Figure 4). Approximately 50 % of the F1 littermates were identified as transgenic. Thus, the gene construct was transmitted according to the Mendelian rules of inheritance. The three transgenic founder lines were viable and fertile.

The production of the GFP transgenic mice was through germline random integration. In this way the transgenic expression pattern would likely be reproducibly observed in F1 and subsequent generations. This approach may eliminate the problem of mosaic expression and, in most cases, the transgenic lines will have correct patterns of expression, mimicking those of endogenous uromodulin gene from which the promoter was derived. However, variable transgenic expression could still occur among different transgenic lines, attributed to a chromosomal position effect [14]. To address this problem, efforts have been made in transgenic animals by using certain chromosomal controlling elements, such as locus control region from the globin gene cluster [15,16] and matrix attachment region from the chicken lysozyme gene [17]. The inclusion of the two copies of the upstream sequence of the chicken β-globin gene, used as an insulator [15], might have stabilized the GFP expression in kidney of the transgenic mice developed in this study.

### Expression of the GFP transgene in tissues of transgenic mice

**Figure 5:**
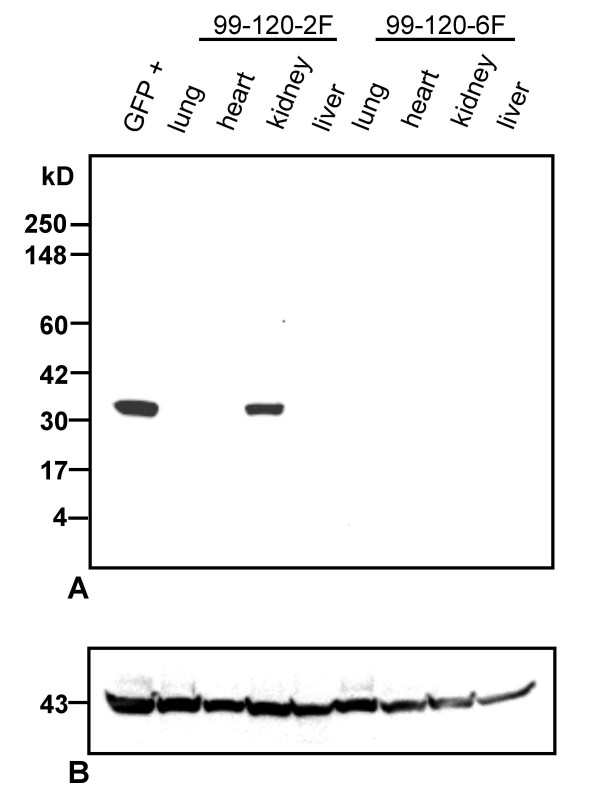
**Western blot analysis of protein extracts from tissues of GUE-GFP transgenic mice. **Tissue extracts from a transgenic mouse (99-120-2F) and a negative control mouse (99-120-6F) were separated on 4–20 % SDS-PAGE and transferred onto the membrane. The GFP positive control was from a cell line with GFP expression. 12 μg of total protein was loaded in each lane. **A. **Immunoreacting bands were detected using a polyclonal anti-GFP antibody (Clontech). **B**. The same blot was reprobed with a mouse monoclonal anti-actin antibody (Chemicon) to show that equal amount of protein was loaded in each lane.

In order to analyze expression of GFP, extracts from various tissues (lung, heart, kidney and liver, etc.), dissected from a transgenic mouse as well as a negative control, were subjected to Western blotting analysis using a polyclonal anti-GFP antibody (Figure 5). The result demonstrated that the GUM promoter directed kidney specific expression of the reporter gene since GFP expression was identified only in kidney extracts but not in lung, heart and liver.

### Cellular localization of GFP reporter gene in the kidney cells of mice transgenic for GUM-GFP

**Figure 6:**
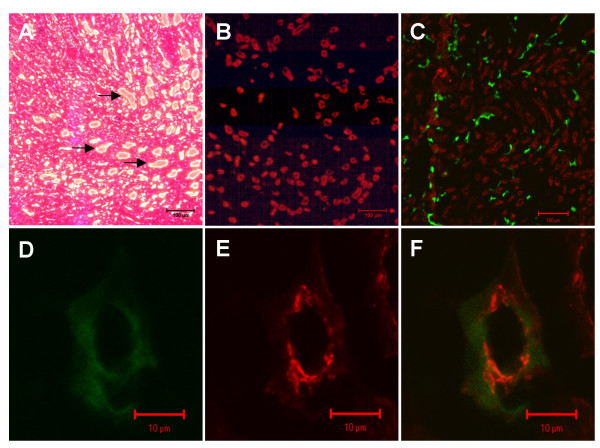
**Demonstration of expression of the goat uromodulin – GFP transgene and endogenous uromodulin in kidney sections by immunostaining. **(**A**). H & E kidney TAL region staining of a control non-transgenic mouse. The arrows show microstructures with thick walls indicative of the thick segment of the ascending limb of Henle's loop within the TAL region. Fixed kidney sections from a negative control mouse (**B**) and the 99-122-1-A5M transgenic mouse (**C**) were stained with a polyclonal anti-human uromodulin antibody, followed by treatment with Texas-Red conjugated secondary anti rabbit IgG antibody. Expression of endogenous uromodulin in the cells (red) appears in a punctuated pattern, whereas the GFP expression is cytoplasmic (green). Immunostaining of similar sections for uromodulin confirmed that expression is restricted to tubular epithelial cells of these structures (**B, C**) indicated by red. The same sections observed for GFP expression by confocal microscopy indicated that expression was restricted to similar structures as in **B **&**C **but not co-expressed with the endogenous uromodulin staining. As expected GFP was absent in the renal sections obtained from a non-transgenic mouse. Both GFP (**D, F**) (green) and uromodulin (**E, F**) (red) were co-expressed in cells in the TAL segment (transgenic mouse, 99-122-1-A5M).

It is established that uromodulin protein is expressed exclusively in the epithelial cells of the TAL of Henle's loop and the early distal convoluted tubule in the kidney [18]. It can, therefore, be used as an ideal marker for identifying these epithelial cells. Since GFP expression in the transgenic mice generated was under the control of the goat uromodulin promoter we reasoned that GFP would co-localize within the same epithelial cells that normally express endogenous uromodulin. Immunohistochemistry analysis of cryo-sections of kidneys dissected from the transgenic mice using an anti-human uromodulin antibody confirmed that expression of the GFP marker protein co-localized with the endogenous uromodulin (red-color stained) in the medulla region of the kidney (Figure 6B, C). It was noteworthy, however, that only a small proportion of the cells within the medulla region of the kidney co-expressed both uromodulin and GFP (Figure 6D–F) with the rest of the cells forming a mosaic pattern between cells expressing either endogenous uromodulin or GFP (Figure 6C). The cellular localization of endogenous uromodulin and transgenic GFP was in the plasma membrane and cytoplasmic space, respectively (Figure 6B, C). The two proteins were not detected in macula densa and from glomeruli, proximal convoluted tubules, thin limbs of the Henle's loops, collecting duct, blood vessels, and the interstitium (data not shown).

### Onset of uromodulin expression during embryonic life

**Figure 7:**
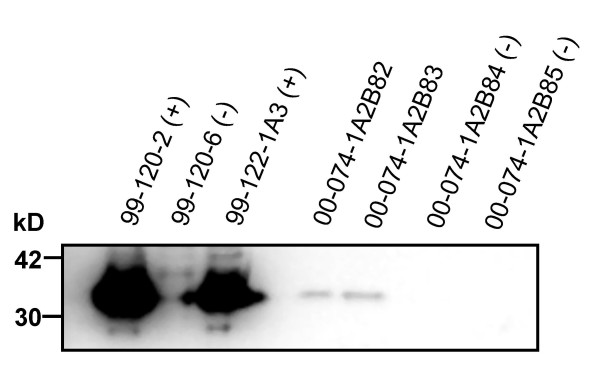
**Western blot analysis of kidney extracts from Day 0 pups, offspring of a GUM-GFP transgenic F2 mouse. **Kidney extracts (12 μg of total protein loaded in each lane) from 99-120-2 and 99-122-1A3 were used as positive controls, whereas 99-120-6 was used as a negative control. GFP immunoreacting protein was detected using the polyclonal anti-GFP antibody (Clontech) in 2 out 4 Day 0 pups, offspring of a transgenic F2 mouse, 00-074-1A2B8M, bred with a wild type mouse.

We investigated the onset of the endogenous uromodulin expression during embryonic life and compared it with the expression of the GFP transgene driven by the GUM promoter as it has been reported that in human the uromodulin protein can be detected after 20 weeks of gestation [19]. Two GUM-GFP transgenic F1 mice were mated with wild type females. Pregnant females were euthanized at days 10–15 of gestation and fetuses were recovered. DNA extracted from the head of each fetus was used to identify by PCR the fetuses as transgenic. The transgenic fetuses as well as negative ones were cryo-sectioned and whole embryo mounts were examined for the presence of the GFP transgene and by immunohistochemistry for uromodulin positive cells. No endogenous uromodulin or GFP positive staining was observed on the embryo sections analyzed, indicating that uromodulin was not detectable during embryonic stage tested (data not shown). However, Western blot analysis performed on the homogenized kidney samples of day 0 pups from a transgenic mouse line demonstrated that the GFP was expressed at birth (Figure 7).

## Discussion

Transgenic animal technology is a valuable tool for understanding gene function. Moreover, the use of transgenic farm animals for the production of pharmaceutically important rc-proteins, such as antibodies, anti-clotting factors, and growth factors, in the mammary gland is also very well documented [20]. A transgenic animal that secretes rc-proteins in its urine offers certain advantages and compliments mammary-gland based rc-protein production system. We have chosen to use the goat uromodulin promoter driving the GFP kidney-specific expression in transgenic mice as a pilot feasibility study for the goat. Compared with other farm animal species, the goat is one of the most promising models for commercial production for rc-proteins and it is used more frequently because of relatively low cost of maintenance and faster breeding times as compared with cattle [21,22]. The feasibility of targeting proteins in the uro-epithelium of bladder and kidney has been demonstrated [9,23-25]. The present study further confirms these studies. The practical outcome of these studies would be to use urine as a bioreactor system in which rc-proteins could be expressed and excreted under the control of the goat uromodulin promoter.

Characterization of targeting the uro-epithelium of bladder and the kidney by the use of the uroplakin II or uromodulin promoters has been recently initiated as mentioned above. Promoters from erythropoietin [26] and rennin [27,28], have been used as kidney specific genetic elements. We report on the tissue specificity of a goat uromodulin promoter in transgenic mice. The reporter GFP gene used was co-expressed in the epithelial cells in the Henle's loop together with the endogenous uromodulin. Using the same GUM-GFP transgene we established the concomitant developmental control of the uromodulin expression and identified that the earliest stage of GFP expression was detected at birth. The tissue specificity observed demonstrate that the goat uromodulin promoter fragment cloned is capable of controlling kidney specific expression of GFP.

It has been reported that small quantities of uromodulin/THP can be detected in blood using sensitive immunoassays [29,30], but it is possible that some proteins in the serum simply cross-react with the antibody raised against the uromodulin/THP proteins [31]. The renal environment appears not to be essential for transcription of the uromodulin/THP gene or synthesis of the protein. Support of this stems from the observation that tissue of rat renal cortex and medulla, when transplanted to the anterior chamber of the eye, proliferated to form tubule-like structures [32]. Sections of these implants reacted with monoclonal antibodies specific for rat uromodulin/THP by immunohistochemistry [18]. The uromodulin/THP immuno-cross-reactive material in liver and cerebrospinal fluid is suggestive that this protein may be present extrarenally in all tissues involved in active chloride transport (such as intestinal and liver, etc), however this finding has not been confirmed either with the use of monoclonal antibodies or the mRNA presence in these tissues [2]. In agreement with this, our data, as well as other recent reports [11,23-25], clearly demonstrate that the uromodulin promoter is an excellent candidate for driving foreign proteins expressed specifically in the kidney.

GFP fluorescence provides an easily visualized marker for the epithelial cells in whole kidneys dissected in frozen sections. This eliminates the need to stain with lectins or antisera to visualize the cells, and therefore allows the cells to be visualized simultaneously with other structures in the developing kidney by combined immunohistochemistry/GFP detection. The arrays of the GFP expression in the kidney cells are of interest (Figure 6). Normally one would assume that since the goat uromodulin promoter was used to drive the GFP expression the same cells should express both uromodulin and GFP all together, however this was only observed in a small proportion of the cells. Due to the copy numbers of the uromodulin promoter integrated in the genome of the transgenic mice it is possible that it competes with the endogenous uromodulin promoter for the transcriptional regulatory factors in the same cells and as a result it may compromise endogenous uromodulin expression. A similar example has been reported for expression of a *Bombyx *cytoplasmic actin gene in cultured *Drosophila *cells to show that expressions of endogenous and recombinant actin genes are not independent [33]. When the *Bombyx *cytoplasmic actin A3 genes were introduced into *Drosophila *cells, the amounts of transcripts from the endogenous cytoplasmic Act 5C and Act 42A actin genes decreased proportionally. Furthermore, in cell lines with stably integrated A3 genes, the increased accumulation of endogenous cytoplasmic actin mRNAs is accompanied by a decrease in the A3 mRNA accumulation: thus when the transcription of resident genes is stimulated, the expression of the transgene is reduced. This balance suggested a competition for the factors involved in the transcription of the cytoplasmic actin genes.

The appearance of uromodulin/THP coincides developmentally with maturation of the TAL of Henle's loop [18]. It has been reported that it is present at eight weeks in the human fetal kidney, two days pre-term in rat [34], and three days pre-term in hamster kidney [35]. Urine from fetal kidney contributes to amniotic fluid from 12 to 14 weeks of gestation [36]. While uromodulin/THP has been described in human amniotic fluid near term by several researchers, studies in mid-term amniotic fluid have been more equivocal [37-39]. Kumar *et al*. have tested amniotic fluid from four patients at 16 weeks and could not detect uromodulin/THP by an enzyme-linked immunosorbent assay in which the lower limit of detection was 20 ng/mL [18]. The earliest stage in which we were able to detect GFP expression driven by uromodulin promoter in transgenic mice was at birth (Figure 7). Although immunohistochemistry was attempted to examine the expression of both uromodulin and GFP at earlier embryonic stages neither could be detected (data not shown). It is likely that the expression level was not high enough to be visualized by the method employed, or that there is a species difference in the ontogeny of the uromodulin protein.

## Conclusion

The goat uromodulin gene promoter efficiently targeted expression of GFP in the kidney of transgenic mice and more specifically in the epithelial cells of the TAL of Henle's loop. Expression of GFP was co-localized in cells expressing endogenous uromodulin. Expression appeared to be specific to kidney at least among the tissues tested.

## Methods

### Cloning of the goat uromodulin gene promoter and its partial 3' end

PCR was performed using genomic DNA from a standard Saanen goat as template with two primers deduced from the conserved regions of human and bovine uromodulin gene promoters [7]. The sense primer was 5' CAT TCT CAG CTC CTY TCY TGC 3' and the antisense primer was 5' AGA GAC CCC AAA TGA TTG ACA 3'. A 350 bp PCR fragment was obtained and sequenced, showing 95 % identity with the known bovine uromodulin promoter sequence [7]. The gene-specific primers were designed from the PCR product to conduct PCR genomic walking using a goat (standard Saanen breed) genomic DNA library, created with the Universal Genome Walker Kit (Clontech). A 3.7 kb PCR fragment was obtained both from the genome-walking library and the goat genomic DNA with newly designed gene-specific primers. This fragment contains a 1.5 kb 5' flanking region, exon 1, intron 1, exon 2 and part of intron 2. Similar strategy was employed for cloning a 2.7 kb fragment of the 3' end. The initial set of degenerate primers was designed based on the conserved regions of bovine uromodulin gene.

### GUM/GFP plasmid construction

A 1.5 kb fragment of the goat uromodulin promoter was subcloned into the pGEM-T easy vector (Promega) and a HindIII-PstI digested promoter insert was ligated with HindIII-PstI digested CMV-IE and EF-1alpha-less pCEEGFP [40] to generate pGUMGFP3. Two copies of a 1.2 kb fragment of the chicken β-globin insulator [15], was digested with XhoI and SalI, and ligated at the XhoI site, located upstream of the goat promoter fragment in pGUMGFP, resulting in the final construct, pING32. The cloned 2.7 kb fragment of the goat uromodulin gene 3'end was not part in the DNA construct used in the generation of transgenic mice.

### Production of uromodulin-GFP transgenic mice

The plasmid backbone of pING32 was removed by XhoI-SphI digestion and the 5.5 kb uromodulin-GFP transgene fragment (Figure 3) was gel-purified and microinjected into the pronuclei of FVB mouse zygotes using standard techniques [41]. The production and maintenance of transgenic mice were conducted at the McIntyre Transgenic Core Facility of McGill University. Animal studies were carried out in accordance with the Guide for the Care and Use of Laboratory Animals as adopted by the U.S. National Institutes of Health.

### PCR analysis

Transgenic mice were detected by PCR analysis of tail DNA with primers amplifying sequences of the insulator, the junction sequence between the uromodulin promoter and GFP. The sense and antisense primers used to amplify the insulator fragment were 5' AGG AGC ACA GTG CTC ATC CAG ATC 3' (Acb347) and 5' GAC GCC CCA TCC TCA CTG ACT 3' (Acb478). The sense and antisense primers for amplifying the uromodulin promoter-GFP were 5' GAT CAT TGG AGG AGA GAT TGC CAG TG 3' (Acb504) and 5' GTC TTG TAG TTG CCG TCG TCC TT 3' (Acb412) (Figure 3). PCR was performed using the Ready-to-Go™ PCR beads (Amersham-Pharmacia Biotech) under the following conditions: 94°C/2 min, 36 cycles of 94°C/30 s, 60°C/45 s, 72°C/45 s and finally 72°C/5 min in a PTC-100™ Programmable Thermal Controller (MJ Research, Inc.). The PCR products were visualized on a 1 % agarose gel and analyzed by FluorChem™ 8000 Advanced Fluorescence, Chemiluminescence and Visible Light Imaging System (Alpha Innotech Corporation).

### Southern blot analysis

Genomic DNA extracted from mouse tail was digested with AflII, subjected to electrophoresis on a 1 % agarose gel and transferred to a Nylon membrane (Roche), positively charged. The membrane was hybridized using a DIG Easy Hyb buffer (Roche) at 42°C overnight containing a PCR-generated probe, labeled by the PCR DIG probe synthesis kit (Roche). This fragment was amplified using the Acb504/412 set of primers amplifying a 490 bp fragment. The membrane was washed once at room temperature with 2 × SSC, 0.1 % SDS for 5 min, three times with 0.5 × SSC, 0.1 % SDS at 68°C for 15 min per wash. Detection of hybridization signals was by using the CDP-Star™ substrate (Roche). The membrane was analyzed by the FluorChem™ 8000 System (Alpha Innotech Corporation).

### Fluorescent In Situ Hybridization (FISH)

The method used essentially was as described [42,43]. Briefly, R-banded chromosomes spreads were prepared by synchronization of goat peripheral blood lymphocytes cultured in RDG medium with thymidine for 18 h. A DNA probe including the 1.5 kb goat uromodulin promoter fragment was labeled with a Bionick labeling kit (Life Technologies Inc). Hybridization medium containing biotinylated probe and genomic goat DNA, was placed on each slide and covered with a plastic film. The slides were incubated overnight at 37°C in a humidified chamber with 50 % (v/v) formamide/2 × SSC followed by wash steps. The slides were incubated for 45 min at 37°C with rabbit anti-biotin Enzo (1 % in modified PBS: PBS with 0.1 % Tween™ 20 and 0.15 % BSA added), and rinsed twice in modified PBS. The samples were incubated with a mouse anti-rabbit biotin-conjugated antibody (0.75 % in modified PBS, Pierce) and rinsed. Slides were stained with Propidium Iodide, mounted and covered with a coverslip. Samples were observed under an Olympus microscope B × 40 using an appropriate combination of filters for fluorescein labeling.

### Western blot analysis

Tissue extracts from kidney, lung, heart, liver and bladder dissected from uromodulin-GFP transgene positive mice as well as negative controls were prepared by homogenization in lysis buffer (50 mM Tris-HCl, 150 mM NaCl, 1 mM EDTA, 1 mM DTT, pH 7.5) containing a tablet of a proteinase inhibitor cocktail (Roche). Proteins recovered in the supernatants following a centrifugation step were separated by SDS-PAGE on a 4–20 % Tris-Glycine polyacrylamide gel (precasted, Invitrogen) and electrophoretically transferred to an ECL nylon membrane (Amersham). After pre-incubation of the membrane for 1 h in TBST [0.24 % (w/v) Tris, 0.8 % (w/v) NaCl, pH 7.6, 0.05 % (v/v) Tween™ 20] containing 5 % nonfat milk, it was incubated in TBST containing a polyclonal anti-GFP antibody (Clontech) at a 1:100 dilution, followed by incubation in TBST containing a HRP-conjugated secondary anti-rabbit IgG antibody (Promega) at 1: 5,000 dilution. Immunocomplexes were detected with the ECL chemiluminescence detection kit (Amersham). Analysis of the immunoreactive bands on the blots was performed with FluorChem™ 8000 System (Alpha Innotech Corporation).

### Immunohistochemistry

Mice transgenic for the uromodulin-GFP transgene, as well as a negative control, were perfused with 4 % paraformaldehyde for fixing the GFP in situ. Kidney and other tissues were dissected from the perfused mice and cryo-sectioned with a thickness of 5 μm. Following incubation of the tissue sections with 5 % goat serum in PBST [PBS, pH 7.4, 0.05 % (v/v) Tween™] they were incubated in PBST containing a polyclonal anti-human uromodulin antibody (BTI) at 1:200 dilution, followed by treatment with a Texas-Red conjugated secondary anti rabbit IgG antibody (Molecular Probe). The sections were mounted with DAKO fluorescence mounting medium (DAKO), and observed with an Olympus UV-microscope with a FITC filter (for GFP) and rhodamine filter (for Texas-red).

### Confocal fluorescence microscopy

Immuno-stained slides were imaged using a Zeiss LSM 410 confocal scanning laser microscope equipped with argon-krypton and helium-neon lasers and appropriate filter sets for independent detection of FITC and Texas Red. Superimposition of serial sections was used to compare the distribution of each of the labels.

## Authors' contributions

YJH carried out the molecular cloning studies, participated in the sequence alignment, analysis of tissues and extracts, and drafted the manuscript. NC participated in the production of the transgenic mice. ASB conducted the FISH studies. JFZ and AL participated in the cloning and the production of the transgenic mice. CNK envisioned and supervised all the studies, as well as drafting the manuscript.
